# Dietary Factors and Prostate Cancer Development, Progression, and Reduction

**DOI:** 10.3390/nu13020496

**Published:** 2021-02-03

**Authors:** Michał Oczkowski, Katarzyna Dziendzikowska, Anna Pasternak-Winiarska, Dariusz Włodarek, Joanna Gromadzka-Ostrowska

**Affiliations:** Department of Dietetics, Institute of Human Nutrition Sciences, Warsaw University of Life Sciences (SGGW-WULS), 159c Nowoursynowska Street, 02-776 Warsaw, Poland; katarzyna_dziendzikowska@sggw.edu.pl (K.D.); anna_pasternak_winiarska@sggw.edu.pl (A.P.-W.); dariusz_wlodarek@sggw.edu.pl (D.W.); joanna_gromadzka_ostrowska@sggw.edu.pl (J.G.-O.)

**Keywords:** prostate cancer, saturated fatty acids, meat, dairy products, vitamin D, vitamin E, selenium, zinc, resveratrol, flavonoids, lycopene

## Abstract

Due to the constantly increasing number of cases, prostate cancer has become one of the most important health problems of modern societies. This review presents the current knowledge regarding the role of nutrients and foodstuff consumption in the etiology and development of prostate malignancies, including the potential mechanisms of action. The results of several in vivo and in vitro laboratory experiments as well as those reported by the clinical and epidemiological research studies carried out around the world were analyzed. The outcomes of these studies clearly show the influence of both nutrients and food products on the etiology and prevention of prostate cancer. Consumption of certain nutrients (saturated and trans fatty acids) and food products (e.g., processed meat products) leads to the disruption of prostate hormonal regulation, induction of oxidative stress and inflammation, and alteration of growth factor signaling and lipid metabolism, which all contribute to prostate carcinogenesis. On the other hand, a high consumption of vegetables, fruits, fish, and whole grain products exerts protective and/or therapeutic effects. Special bioactive functions are assigned to compounds such as flavonoids, stilbenes, and lycopene. Since the influence of nutrients and dietary pattern is a modifiable risk factor in the development and prevention of prostate cancer, awareness of the beneficial and harmful effects of individual food ingredients is of great importance in the global strategy against prostate cancer.

## 1. Introduction

Over the past few decades, non-communicable chronic diseases have become the main health problem of people worldwide. The results of epidemiological studies indicate cancers as the second most common cause of death after cardiovascular diseases. A significant increase in the number of patients diagnosed as having cancer is particularly observed in developed and developing countries. Prostate cancer is one of the most common cancers in men [1,2]. According to the epidemiological data, prostate cancer is classified as the second-most common cancer, after lung cancer, and the sixth cause of death due to malignant neoplasms among men worldwide, with an estimated number of 1,276,000 new cases and 359,000 deaths in 2018 [2]. The highest incidence of prostate cancer is observed in highly developed countries [3], mainly in the USA, Canada, Australia, and European Union countries [4,5]. It is estimated that the incidence of prostate cancer will increase to nearly 2.3 million new cases by the year 2040 and that the death rate will reach 740,000 [2].

An increasing number of prostate cancer cases makes it necessary to study its etiology and to develop strategies for its prevention. Furthermore, increasing attention is being paid to the role of modifiable risk factors, including lifestyle and diet in particular, which are associated with prostate cancer risk. The Western-type dietary pattern (characterized by high consumption of processed food, meat, and meat products with high fat content, and a lower intake of fruits and vegetables), in contrast to the minimally processed food items, is considered to be associated with a higher risk of cancer incidence [6]. Yahya et al. [7] in a case–control study identified two major dietary patterns: Western and Mediterranean. Their findings suggest that the Western dietary pattern may increase prostate cancer risk, while the Mediterranean dietary pattern may be beneficial. Additionally, a case–control study conducted in Argentina [8] that enrolled 147 prostate cancer patients cases and 300 controls identified four dietary patterns: ‘Traditional’ (high consumption of fatty red meats, offal, processed meat, starchy vegetables, added sugars and sweets, candies, fats, and vegetable oils), ‘Prudent’ (non-starchy vegetables and whole grains), ‘Carbohydrate’ (sodas/juices and bakery products), and ‘Cheese’ (cheeses). The results of the Niclis et al. [8] study showed high adherence to the traditional and carbohydrate patterns, promoting prostate cancer development. These observations have been confirmed in other studies [9]. Shin et al. [10] also suggest that a Western-style diet may contribute to a higher prostate cancer risk, whereas the prudent dietary pattern is associated with lower risk.

The knowledge of mechanisms linking nutrient action with carcinogenesis modulation and interactions between them is crucial for designing the health programs of societies of highly developed countries. A well-balanced diet provides bioactive compounds with an emerging potential to reduce prostate cancer risk and can be helpful in prostate cancer therapy [11,12,13]. In this review, the association between prostate cancer development and chosen dietary factors is shown. Additionally, the review provides a summary of the current knowledge on the effects of nutrients and foodstuffs in the etiology and prevention of prostate cancer and highlights the protective effect of bioactive substances, such as vitamin E and tocopherols, lycopene, and selected flavonoids.

## 2. Prostate Cancer—Etiology and Pathogenesis

Although the etiology of prostate cancer is still not well known, there are some factors that have been shown to be related to an increased risk of prostate cancer development. Among them, age, family history, ethnicity [14], and genetic factors [15] are the major non-modifiable factors [16]. Additionally, smoking, being overweight and obesity, a sedentary lifestyle, and poor quality of diet are the other important factors that are positively correlated with the risk of prostate cancer [17]. However, the mechanisms underlying the individual role of these factors and prostate cancer development remain unclear. The results of recent studies emphasize the role of epigenetic changes associated with prostate cancer. Complex interactions between genetic and environmental factors result in the deregulation of genes controlling epigenetic processes involved in histone modifications, DNA methylation, and noncoding miRNA [18].

Compared to other cancers, the risk of prostate cancer increases dramatically with age. Brawley [19] demonstrated that only 0.6% of patients aged 35–44 years were diagnosed with prostate cancer, but the prevalence of cancer in those aged 65–74 years was much higher (35%). Malik et al. [20] analyzed the age-dependent relationship of prostate cancer risk and observed that the number of cancer cases in men over 80 years of age was approximately 40-fold higher compared to men under 50 years of age. The results of many studies analyzing the race as a predictor of prostate cancer show contradictory conclusions. A multi-cohort study of 306,100 patients with prostate cancer did not find any significant association between black ethnicity and the disease [21]. However, Nettey et al. [22] revealed that the frequency of prostate cancer was high in the black population compared to the non-black population. The results of a meta-analysis by Adeloye et al. [23] showed a median incidence rate of prostate cancer of 19.5/100,000 in a population from the African region, which was higher when compared to Asian [15], European [24], or American populations. Additionally, the results obtained by Kelly et al. [25], based on the analysis of data from registry databases (2004–2014) covering 28% of the US population, showed the highest incidence rate of prostate cancer (38.3/100,000 person-years) in the African-descent population compared to white (17.3), Hispanic (18.9), or the Asian-American population (11.2/100,000 person-years).

Almost all prostate cancers are formed from glandular tissue (95% are adenocarcinomas), while other histological types of these cancers are rare [26]. Prostate cancer is multifocal and genetically heterogeneous, and its foci are often formed independently in different areas of the gland [27]. In more than half of the cases, the cancer is found in the posterior part of the gland. It should be noted that a characteristic feature of prostate cancer is the long latency period of several years, during which prostatic intra-epithelial neoplasia develops [28]. 

The exact molecular mechanisms responsible for prostate cancer are not fully understood. However, it is known that changes in the expression of growth factors, such as a transforming growth factor-β (TGFβ), vascular endothelial growth factor (VEGF), or insulin-like growth factors (IGFs), especially IGF-1, may promote the development of this disease [29]. The prostate is an organ with high hormonal sensitivity, and its biological functions are stimulated under the influence of hormones [30,31]. In addition, the etio-pathogenesis of prostate cancer depends, among others, on the steroid hormones (androgens and estrogens) [32,33] and IGFs [34]. In about 70% of cases, the initial development of prostate cancer is influenced by male steroid hormones [35]. Sex steroids contribute to regulating the proliferation of prostate epithelial cells. Testosterone and its metabolites affect the expression level of proto-oncogenes, which refer to the genes encoding the receptors of various growth factors and those encoding serine proteases, including prostate-specific antigen (PSA) and protein C [36]. Through the changes in the activity and expression of cell cycle regulatory proteins, androgens can potentially stimulate cell proliferation. Testosterone can also modulate apoptotic processes in hormone-dependent prostate cells by affecting the expression level of apoptotic genes and, thus, promoting cancer development [35]. Prostate cancer is a hormone-dependent neoplasm, and its cells secrete a PSA, in which the level is, in most cases, correlated with the stage of cancer development [37].

Estrogens and their receptors (ERs), as described by Dobbs et al. in their comprehensive review [32], play a significant role in the development and progression of prostate cancer. Membrane-associated ERs (ER-α and ER-β) and G protein-coupled receptor 30 (GPR30), as well as nuclear ERs, are present in the prostate gland cells. In particular, the activation of ER-α is believed to contribute to prostate carcinogenesis, whereas GPR30 appears to be crucial for mediating the nongenomic action of estrogens by mitogen-activated protein kinase pathways. These impacts might be associated not only with the receptor-mediated effects of estrogens but also with their DNA-damaging and potentially mutagenic activity and subsequent carcinogenesis. Moreover, many nutrients affect prostate hormonal regulation, which is discussed in Section 3.1.2 of this review. 

## 3. The Role of Nutritional Factors and Foodstuffs on Prostate Cancer Risk

Epidemiological and observational studies demonstrate an association between an unhealthy diet, excessive alcohol consumption, tobacco smoking, and a higher risk of chronic non-communicable chronic diseases [38], including cancers [39,40,41]. The relationship between nutrition and carcinogenesis has been thoroughly studied since the 1970s. An excessive energy intake frequently linked with a high intake of saturated fatty acids (SFAs) and sugars [42,43,44] and a low intake of complex carbohydrates [45], polyunsaturated fatty acids (PUFAs) [46], fruits, and vegetables [47] is associated with a higher risk of various cancers, including prostate cancer. Additionally, certain food products (such as processed, smoked meat products) can be a source of carcinogenic compounds (polycyclic aromatic hydrocarbons, N-nitroso compounds, and heterocyclic aromatic amines), which can initiate and promote prostate carcinogenesis [48].

### 3.1. Nutrients and Dietary Patterns

#### 3.1.1. Excessive Consumption of Selected Nutrients

Long-term excessive energy supply contributes to obesity and other negative health outcomes, such as cardiovascular diseases and cancers [49]. The results of both epidemiological and laboratory studies indicate that the energy value of the diet and the identified mediators, which are significantly influenced by the energy supply, play an equally important role in the development of prostate cancer [50]. The results of many extensive research (randomized controlled studies and meta-analyzes) report the link between obesity and the risk of prostate cancer, but, in some cases, are inconsistent and vary depending on the stage of disease and investigating parameters (cancer diagnosis, progression, or aggressiveness). The results of the REDUCE study [51] conducted among 6427 men aged 50–75 years (of whom 1304 men were obese and 1448 participants were diagnosed with prostate cancer) indicated that obesity was positively correlated with high-grade cancer cases scored at a Gleason score of ≥7 (OR = 1.28, 95% CI = 1.01–1.63). Similarly, the results from meta-analysis of 17 cohort studies [52] with 3,569,926 individuals with the median follow-up time ranged from 2 to 49 years indicated that obesity was not significantly associated with total prostate cancer risk, but obese men were characterized with a significantly higher risk of aggressive prostate cancer (OR = 1.14, 95% CI = 1.04–1.25), as well as with a higher risk of prostate cancer-associated mortality (OR = 1.24, 95% CI = 1.15–2.33). On the other hand, the recently published meta-analysis of Harrison et al. [53] based on 18 studies and 1,146,847 participants demonstrated a significant effect of increase in body mass index (BMI) (for every 5 kg/m^2^) on advanced prostate cancer risk (HR = 1.06, 95% CI = 1.01–1.12). The observations made by Langlais et al. [54] in groups of 5200 patients diagnosed with prostate cancer, of whom 685 experienced the clinical symptoms of recurrence, revealed that obese (with BMI = 30–35 kg/m^2^) and very obese (with BMI greater than 35 kg/m^2^) patients were at higher prognostic risk at diagnosis (OR = 1.5, 95% CI 1.2–1.8 and OR = 1.7, 95% CI = 1.2–2.3, respectively) and were more likely to diagnose an advanced prostate tumor stage between biopsy and surgery (OR = 1.3, 95% CI = 1.0–1.6 and OR = 1.6, 95% CI = 1.1–2.1, respectively) when compared with patients with normal Body Mass Index (BMI). Finally, the very obese patients were also at a significantly higher risk of recurrence compared to normal weight patients (HR = 1.7, 95% CI = 1.1–2.5). Preclinical studies indicate that weight loss suppresses the growth of prostate cancer by reducing blood levels of IGF-1 and reducing inflammation accompanying obesity [55]. Moreover, it was found that the degree of energy reduction in the diet has a greater impact than its source [56].

Reduction in the energy supply in the diet modulates numerous processes occurring in tissues and cells, which may inhibit carcinogenesis. One of the most important effects is the enhancement of the apoptosis rate relative to angiogenesis and cell proliferation. These processes are more enhanced in tissues affected by cancer, and the ratio of proliferation to apoptosis is many times higher than that of normal cells. Numerous studies suggest the beneficial effects of a reduced energy supply, which include enhanced programmed cell death processes [57]. Additionally, energy restrictions have the most beneficial effects of inhibiting the progression of the existing cancer [58,59].

The main mediator of prostate carcinogenesis dependent on the dietary energy balance is IGF-1. A high level of insulin observed in obesity results in reduced plasma concentration of insulin-like growth factor-binding proteins (IGFBP) and sex-steroid-binding globulin (SHBG), including testosterone and its metabolites, which is mainly dihydrotestosterone (DHT) in men. Somatomedins, mainly IGF-1, enhance proliferation and angiogenesis and inhibit apoptosis in the presence of DHT because of an increased level of IGFs [60,61]. In vitro studies have revealed a decrease in IGF-1 concentration and an inhibition of neoplastic cell growth when the energy supply was limited [62,63]. It has also been demonstrated that the benefits resulting from energy restrictions are lost in the case of a simultaneous increase in the concentration of IGFs, which directly proves the role of these mediators in the processes of carcinogenesis resulting from excess energy. An unbalanced diet in terms of energy, due to increases in IGF-1 concentration, may also enhance the synthesis of VEGF, which is involved in angiogenesis. The formation of new blood vessels allows the neoplasm to grow and increase its malignancy by gaining the ability to metastasize. Reduced energy intake also increases the level of activity of antioxidant enzymes with a protective effect, which can reduce the oxidative damage of proteins [64].

In addition to the energy efficiency of the diet, dietary patterns are an important issue. Ambrosini et al. [6] using a population-based case–control study in Australia stated that the Western diet may increase the risk of prostate cancer, especially the development of its aggressive form. Patients diagnosed with such aggressive cancers were shown to have a reduced risk of total mortality and prostate-cancer-related mortality when adhering to the prudent pattern, which is associated with a high consumption of vegetables, fruits, fish, legumes, and whole grain products. Results of an Australian cohort study on 15,000 men aged 34–75 years showed that, in the case of the Western dietary pattern, patients consuming high levels of processed and red meat, high-fat dairy, and whole milk products had a higher risk of total mortality and prostate-cancer-related mortality [65,66]. Results of another case–control study on 40–80-year-old men from Jamaica showed that a diet particularly rich in refined carbohydrates was positively associated with the risk of prostate cancer [67]. On the other hand, a population-based multi-case control study from seven Spanish provinces showed that patients on Mediterranean and DASH (Dietary Approaches to Stop Hypertension) diets had a lower risk of aggressive prostate cancer [68]. Additionally, these observations were similar for both African-Americans and Americans of European origin who showed that the higher diet quality represented by the Mediterranean or DASH diets may reduce the risk of aggressive prostate cancer [69]. One common element in the Mediterranean, Prudent, and DASH diets is a large proportion of vegetables. These products are rich in both antioxidants and other nutrients, e.g., oleic acid [70], which have anti-cancer properties. Moreover, they are mainly products with low energy density, which has a positive effect on general nutritional status. The risk of carbohydrate metabolism disorders, including insulin resistance, is reduced in body weight normalization, which is also important in terms of reducing prostate cancer risk. However, as documented by Cheng et al. [71], in a large meta-analysis of 10 observational studies, the Mediterranean dietary pattern had no relationship with prostate cancer risk. A case–control study on 60 newly diagnosed and 60 hospital-based controls from Iran demonstrated a high association between Western diet consumption and prostate cancer risk, whereas the Mediterranean dietary pattern did not reveal a significant inverse association [7].

#### 3.1.2. Dietary Fat

The association between fat intake and prostate cancer risk has been extensively studied for many years and still remains controversial. Dietary fat intake, depending on its quality and quantity, can result in a two-fold increase in the risk of individual stages of prostate cancer. The NIH-AARP Diet and Health Study [72], which enrolled 288,268 men and 23,281 prostate cancer cases with an observation period of nine years, found that a high intake of SFAs (at least 13.3% of total energy intake) compared with individuals consuming SFAs at the lowest level (below 5.8% of total energy) were characterized by a higher risk of advanced or fatal prostate cancer cases (highest vs. lowest quintile HR = 1.21, 95% CI = 1.00–1.46 and HR = 1.47, 95% CI = 1.01–2.15, respectively). Furthermore, the authors reported that a higher intake of n-3 α-linolenic acid (ALA) (0.88% of total energy intake) compared with intake at the level of 0.41% was associated with a higher risk (HR = 1.17, 95% CI = 1.04–1.31) of advanced prostate cancer. At the same time, the patients who consumed a higher amount of n-3 eicosapentaenoic (EPA) acid (0.018% vs. 0.003% of total energy intake) experienced a lower risk of fatal prostate cancer (intake of highest vs. lowest quintile intake: HR = 0.82; 95% CI = 0.64–1.04). As shown in the recently published prospective SABOR (San Antonio Biomarkers of Risk) cohort study [73], covering a group of 1903 men, of whom 229 were diagnosed with prostate cancer and with a median follow-up of 8.9 years, the relative risk of prostate cancer was associated with an intake of dietary fat, especially with an increasing intake of SFAs (HR = 1.19; 95% CI = 1.07–1.32) and trans fatty acids (TFAs) (HR = 1.21, 95% CI = 1.08–1.35). The associations still remained significant after comparing the data between the highest and lowest quintiles of fat intake (HR = 1.77, 95% CI = 1.04–3.01 and HR = 2.24, 95% CI = 1.34–3.75, respectively). The composition of fatty acids, rather than the total dietary fat intake, plays an important role in prostate cancer progression and development. The results of the study conducted by Ohwaki et al. [74], using data coming from 13,594 men aged >50 years, indicated that the serum prostate-specific antigen (PSA) level was 3.4% (95% CI = 0–6.9%) higher in those men who consumed more than 31.2% of energy from fat, compared to the ones consuming below 20.1% of energy from fat. 

There is convincing evidence from basis and population-based studies that intra-prostatic inflammation contributes to prostate carcinogenesis [75], and obesity also provides a pro-inflammatory state in an organism [76]. Many studies suggest that inflammation plays a crucial role in particular stages of prostate carcinogenesis (initiation, promotion, malignant transformation, invasion, and metastasis) as well as cancer progression [77]. A study by Gurel et al. [78] demonstrated a close relationship between prostate inflammation and cancer development. Men who had a biopsy core with inflammation revealed a 1.78-fold (95% CI = 1.04–3.06) higher chance of developing prostate cancer than men whose prostate biopsy cores did not show inflammation. 

A report from the prospective Health Professionals Follow-up Study (1986–2010) [79] of 4577 men aged 40–79 years with non-metastatic prostate cancer found that the replacement of 10% dietary carbohydrate energy by plant fat was correlated with a reduction in the risk of lethal prostate cancer (HR = 0.71, 95% CI = 0.51–0.98), as well as all-cause mortality (HR = 0.74, 95% CI = 0.61–0.88), while the replacement of 1% or 5% dietary carbohydrate energy by saturated fatty acids (SFAs) was correlated with an increased overall mortality rate (HR = 1.25, 95% CI = 1.05–1.49 and HR = 1.30, 95% CI = 1.05–1.60, respectively). Allott et al. [80], based on the results from population-based North Carolina-Louisiana Prostate Cancer trial (data from 1854 individuals with prostate cancer, of whom 321 were diagnosed as highly aggressive and 1533 were diagnosed with lowly/intermediately aggressive cancer) also showed a significant relationship between a high consumption of SFAs and the aggressive form of prostate cancer (the highest vs. lowest tertile: OR = 1.51, 95% CI = 1.10–2.06). The results of population-based case–control study [81] with 605 prostate cancer cases and 592 healthy controls aged 40–64 years indicated that a lower energy intake (<1322 kcal/d vs. more than 2439 kcal/d) was associated with a lower (by approximately 50%) risk of local and of regional or distant prostate cancer.

Similar relationships were observed in basic studies conducted on animals, which demonstrated a faster growth of cancer cells following an increased supply of fats. It was also found that a low availability of fat in food inhibits the development of cancer, causing reduced sensitivity of cancer cells to androgens, which significantly affects the degree of cancer malignancy, thereby, prolonging the survival of animals [82].

**Figure 1:**
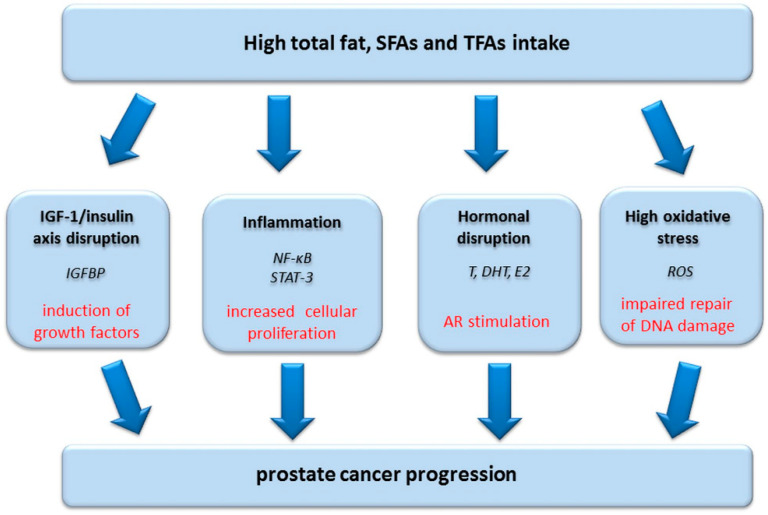
The possible effects of high total fat, saturated fatty acids (SFAs) and trans fatty acids (TFAs) intake on prostate cancer progression. IGFBP: insulin growth factor binding protein (inhibited activity/protein level reduction). NF-κB: nuclear factor kappa B (increased expression). STAT-3: signal transducer and activator of transcription 3 (increased expression). T, DHT, E2: testosterone, dihydrotestosterone, 17β-estradiol (increased secretion). ROS: reactive oxygen species (increased generation). AR: androgen receptor.

The possible mechanisms of the effects of fat intake on prostate cancer carcinogenesis are linked with the fat intake effect on hormonal regulation and androgen levels and include oxidative stress, exposure to toxic pesticides, and specific effects of individual fatty acids. A high-fat diet not only disrupts prostate hormonal regulation and causes oxidative stress and inflammation, but also alters growth factor signaling [83,84] and lipid metabolism [85], which both contribute to prostate carcinogenesis (Figure 1). Moreover, a high-fat diet, similar to a Western-style diet, induces intraprostatic inflammation and cellular proliferation of the mouse prostate gland due to intraepithelial neoplasia and prostate cancer [86]. A high-fat diet also causes a significant increase in proinflammatory cytokines and activates signaling pathways involving two transcription factors: STAT-3 and NF-κB [87].

As described earlier, prostate cancer is a hormone-dependent neoplasm strongly associated with androgen levels. Several studies have shown a relationship between fat intake and a concentration of steroid hormones in serum. Decreased levels of androgens in serum were observed in vegetarians or people who reduced their dietary fat intake. Laboratory studies have also indicated a relationship between high fat consumption and an increased 5-α reductase activity involved in the conversion of testosterone to DHT [88]. In a mouse model study, lower levels of testosterone, 17β-estradiol, and androgen receptors, achieved through the consumption of n-3 fatty acid-rich diet, which promoted apoptosis and suppressed prostate epithelial cell proliferation [89].

Several population-based epidemiological studies have confirmed that the fatty acid profile in the diet is more important than the total amount of fats [90]. A significant reduction in the risk of an advanced stage of prostate cancer with metastasis was found with an increased consumption of fish, especially fatty ones. A two-fold lower relative risk was observed in people eating fish more than three times a week compared to those eating fish less than two times a month. In Sweden, the risk of prostate cancer in men who do not consume fish was also more than double when compared to those who eat them in average amounts [91]. The results from a 22-year prospective study [92] (382,144 person-years, 2161 men with prostate cancer) reported that, although fish intake was not significantly related to prostate cancer risk, the participants diagnosed with prostate cancer who consumed fish more than five times per week had a significantly (by approximately 50%) lower risk of prostate cancer death compared with those who consumed fish less than once per week (RR = 0.52, 95% CI = 0.30–0.91). Similarly, the protective association between high fish consumption and lower prostate cancer-specific mortality risk was demonstrated in a meta-analysis by Szymański et al. [93], which included four cohort studies (with 49,661 men and 740 fatal prostate cancer cases).

Among PUFAs, linolenic, EPA, and docosahexaenoic (DHA) acids reduce the overall risk of prostate cancer and its advanced stages. Alpha-linolenic and myristic acids increase the incidence of advanced prostate cancer. The linoleic to alpha-linolenic acid ratio is also important, and a lower risk is associated with higher values of this ratio [94]. However, Brasky et al. [95] in a case–cohort study (with 834 men with prostate cancer and 1393 sub-cohort participants) noted the potential risk of high consumption of n-3 fatty acids associated with the prostate cancer risk and reported that higher concentration of long-chained n-3 fatty acids in plasma phospholipids (>5.3% vs. <3.7% of total fatty acids) was related to a 44% and 71% higher risk of low-grade and high-grade prostate cancer, respectively.

It should be mentioned that results from randomized controlled trials regarding the effects of supplementation of fish oil (EPA and DHA acids) on prostate cancer prevention have not demonstrated significant findings [96]. Similarly, results obtained from a randomized controlled trial conducted by Zhang et al. [97] among 86 men aged 50–78 years show a lack of a significant effect in Ki-67 protein expression (a marker of proliferation) in prostate tissue in men supplemented with fish oil (1.9 g of EPA and DHA per day for 60 days). A three-month randomized, double-blinded, clinical trial [98] investigating the effects of supplementation of 3 g of fish oil (containing 1,098 mg of EPA and 549 mg of DHA vs. a placebo) in a group of 69 men with low-burden prostate cancer demonstrated no differences in gene expression *IGF-1*, *IGF-1* receptor, and *COX2* in prostate tissue when compared to a placebo. 

When analyzing the influence of different types of dietary fats and their sources, attention should also be paid to TFAs. They are formed in technological processes, such as the hydrogenation and deodorization of vegetable fats. Large quantities of trans isomers are present in hard margarines, and they are also found in small amounts in dairy products [99]. An increased consumption of products containing trans isomers might enhance systemic inflammation and insulin resistance, which are factors that contribute to cardiovascular diseases, type 2 diabetes, and cancer [100,101]. A large epidemiological case–control study [102] (with 1799 prostate cancer cases and 5039 controls) showed a significant positive correlation between the consumption of TFAs and the risk of prostate cancer risk. The participants who consumed more than 19.1 g of TFAs per week were more likely (by 42%) to be at risk of prostate cancer than those who consumed less than 6.9 g/week [102]. Similarly, results from a population-based SABOR (San Antonio Biomarkers of Risk) cohort study [73] showed that the increased intake of TFAs (evaluated as the highest vs. the lowest quintile of intake) was associated with a 2.2-fold higher risk of prostate cancer. In turn, results from another case–control study [103] (with 1012 men, of whom 506 participants were diagnosed with prostate cancer and are of Caucasian and African-American ethnicity), the significant association between higher trans-fatty acid intake (the lowest and highest quintile of TFAs intake: 5.75 g/d vs. <2.52 g/d: OR = 2.77, 95 CI = 1.6–4.8) and advanced prostate cancer was indicated. This significant relationship was observed only in participants of Caucasian descent. This effect was modified by a functional polymorphism in the RNASEL gene (R462Q), which encodes 2-5A-dependent ribonuclease and has been associated with a predisposition to prostate cancer. The positive effect between TFA intake and advanced prostate cancer was enhanced only in Caucasian men with a QQ/RQ genotype.

The quantity and quality of fat consumed are directly related to the frequency of consumption of products, such as dairy or fish. These products are concurrently a source of SFAs, PUFAs, and MUFAs as well as numerous xenobiotics with carcinogenic properties. One of the mechanisms by which xenobiotics exert their influence is the induction of oxidative stress [104]. Oxidative stress is the result of the accumulation of reactive oxygen species (ROS), which participates in many mechanisms of cell growth and development and can damage the DNA. Under oxidative stress conditions, the p-53 suppressor genes show a mutagenic characteristic. Moreover, it was demonstrated that ROS can increase the expression of oncogenes. Fats contained in food causes an increase in the level of oxidative stress markers and are commonly considered to be pro-oxidants. A high supply of dietary fats can also eliminate vitamin E and other antioxidants, such as lycopene and selenium, by activating the antioxidant effect [104].

#### 3.1.3. Meat Consumption

In highly developed countries, meat is one of the main animal-derived products in the diet. It provides significant amounts of protein with a high nutritional value, fats, and vitamins (mainly from the B group), while also serving as a good source of minerals, in particular iron, copper, and zinc, which are easily assimilated. However, the results of a long, prospective, cohort study (The National Institutes of Health (NIH)-AARP Diet and Health Study) [105] conducted among 10,313 prostate cancer patients, of whom 1102 patients were advanced and 419 were diagnosed with fatal disease, showed that participants who consumed more than 66.1 g of red meat per 1000 kcal had an increased (by 12%) risk of all prostate cancer compared to patients who reported red meat intake below 11.6 g/1000 kcal (multivariate HR = 1.12, 95% CI = 1.04–1.21). The association was stronger for advanced disease, with a more than 30% higher risk observed among men consuming meat at significant amounts compared to those consuming small amounts (multivariate HR = 1.31, 95% CI = 1.05–1.65). Moreover, the intake of processed meat was also associated with an increased risk of advanced prostate cancer (consumption of 24.6 g/1000 kcal vs. 2.2 g/1000 kcal: multivariate HR = 1.32, 95% CI = 1.08–1.61) [106,107]. The only meat-cooking method that was associated with an increased risk was grilling/barbecuing, including heme iron, nitrite/nitrate, and benzo[a]pyrene effects [105]. Results from a prospective case–control study by John et al. [108] with 726 prostate cancer diagnosed cases and 527 controls aged 40–79 years indicated that a high consumption of hamburgers, processed meat, grilled red meat, and well-done processed red meat was strongly associated with higher risk (by 79%, 57%, 63%, and 52%, respectively) with advanced prostate cancer risk compared with no processed red meat consumers. A particularly high risk was found with an increased consumption of hamburgers [108]. On the other hand, Kuotros et al. [109] showed in a large cohort US study that an increased incidence of advanced prostate cancer was not associated with the type of meat, but with the overall consumption of meat in a well-baked or very well-baked form, mainly pan-frying or grilling. At the same time, some studies have shown that the consumption of processed meat did not influence the risk of developing prostate cancer [110,111,112]. Finally, the results of a meta-analysis of 19 cohort studies did not show any correlation between a high consumption of red meats and the formation of prostate cancer, and, concurrently, only a slightly increased risk of this disease was shown to result from increased meat consumption [106]. In addition, the results of another meta-analysis of 15 studies of red meat and 11 studies of processed meat were not supportive of an independent, positive association between the intake of red or processed meat and the development of prostate cancer [107]. Another research report indicated that eating white meat is not associated with the risk of this cancer [108].

The revised 2018 report of the World Cancer Research Fund and American Institute for Cancer Research on diet and prostate cancer stated that evidence showing the relationship between the consumption of red and processed meat and prostate cancer are limited and inconclusive [41]. However, a recently published report of data from 172 countries showed a link between higher meat consumption, regardless of the type (red or white), and a higher risk of this cancer. Both red and white meat produce the same carcinogens when they are cooked at high temperature [113]. The inconsistent results regarding the impact of meat consumption on the risk of prostate cancer may be due to differences in the studied populations and their size, and the methods used for processing meat products and for assessing the diet. Therefore, further research is needed to explain this relationship.

Initially, the positive relationship observed between meat intake and prostate cancer in a study was attributed to high energy and the presence of higher amounts of SFAs found in a diet rich in meat products [114]. More detailed research has demonstrated that these factors were not the only promoters of carcinogenesis of this cancer. The disease is now mainly associated with excessive consumption of red and processed meat. This relationship is also supported by the International Agency for Research on Cancer [115]. The association between prostate cancer and meat consumption can be explained in several ways. One of them is that red meat cooked at a high temperature (grilled/barbecued) may contain many carcinogens, such as heterocyclic amines (HAs) and polycyclic aromatic hydrocarbons (PAHs). 

One of the most common HAs in cooked meat products (including grilled and barbecued meat) is 2-amino-1-methyl-6-phenylimidazo[4,5-b]pyridine (PhIP). Shirai et al. [116] revealed that exposure of Fisher344 rats to PhIP (added to the feed at a dose of 400 ppm and administered for 52 weeks) led to prostate carcinoma of the ventral lobe in 18 of 27 animals. Moreover, atypical hyperplasia of prostate epithelial cells was confirmed in ventral (81.5% of cases) and anterior prostate (30% of cases) lobes of animals exposed to PhIP. Hrubá et al. [117] showed that benzo[a]pyrene, which is one of the most carcinogenic PAHs and an activator of aryl hydrocarbon receptor, suppressed the expression of genes involved in cell cycle progression and DNA repair in prostate cancer cells. N-nitroso compounds may be present in processed meat due to the endogenous production from the meat itself or added preservatives. In addition, it has been shown that heme iron has catalytic effects on the formation of genotoxic and cytotoxic aldehydes by lipoperoxidation, and the endogenous formation of carcinogenic N-nitroso compounds [113].

#### 3.1.4. Selenium and Zinc

Selenium and zinc are trace elements essential for the normal functioning of organisms with well-documented roles in immune response [118,119], reduction of oxidative stress, and DNA repair [120]. Both trace elements are also components of numerous enzymes that regulate key biochemical processes in organisms [121].

Selenium content in food is dependent on the level of this trace element in soil. Grains, meat, fish, and dairy products are the main food sources of selenium. The recommended daily intake of this element for an adult male is approximately 70 µg per day [122]. It is known that the soil of most European countries is poor in selenium content. In organisms, selenium is found in the form of selenoproteins, which contain selenocysteine or selenomethionine [123]. Selenium-dependent enzymes present in the human body include iodothyronine deiodinase type I and III, thioredoxin reductase, and antioxidant enzymes, such as glutathione peroxidase (GPx) and superoxide dismutase (SOD), which are elements of the body’s antioxidant defense system [124].

Several prospective studies and meta-analyses indicate a significant relationship between blood selenium concentration and the risk of prostate cancer. Cui et al. [125], based on 17 case–control studies including data from 34,901 participants, reported a significantly lower risk of prostate cancer in men with a higher serum selenium concentration (OR = 0.74, 95% CI = 0.64–0.91, *P* for heterogeneity = 0.001). Similarly, the findings from a meta-analysis by Hurst et al. [126] covering 12 studies with 13,257 participants (of whom 5007 subjects were diagnosed with prostate cancer), demonstrated a lower risk of the disease with increasing selenium concentration in plasma or serum up to 170 ng/mL. At the same time, the participants whose toenail selenium concentration was in the range of 0.85–0.94 µg/g that were characterized by a significantly lower prostate cancer risk (RR = 0.29, 95% CI = 0.14–0.61).

The results of a meta-analysis of 38 studies showed a lower (by 14%) relative risk of prostate cancer in relation to selenium intake, as well as a lower (by 33%) risk of progression to advanced stages of disease [124]. Allen et al. [127] demonstrated a lower risk of aggressive prostate cancer in individuals with high blood selenium concentration (OR = 0.43, 95% CI = 0.21–0.87). It was observed that the risk of prostate cancer among men supplemented with 200 µg of selenium per day was reduced by approximately 65%. The most beneficial effect of diet enrichment with selenium was observed in people under 65 years of age with a low level in the body and a low serum PSA concentration. During aging, a decrease in selenium is observed, which may increase the incidence of prostate cancer [128,129,130]. This was also confirmed in an animal model study. Waters et al. [131] revealed a U-shaped relationship between the selenium status in humans and prostate cancer risk.

The results of a randomized, placebo-controlled trial (SELECT Study) [132] involving a group of 35,533 relatively healthy men aged 50 or older with an overall follow-up of 5.5 years did not reveal a significant effect of supplementation of selenium from *L*-selenomethionine (at a dose of 200 µg/d) alone or in combination with vitamin E (400 IU/d) on prostate cancer prevention when compared with the placebo group. The hazard ratios for prostate cancer mortality for groups of men assigned for selenium and selenium and vitamin E supplementation were 1.04 (99% CI = 0.87–1.24,) and 1.05 (99% CI = 0.88–1.25) when compared with the placebo group, respectively. In turn, the results from a prospective Health Professionals Follow-Up Study [133] with 4459 men diagnosed with nonmetastatic prostate cancer reported during an 8.9-year follow-up showed a two-fold higher crude death rate among men who supplemented selenium (>140 µg/d) compared with non-users. The multivariable analysis also revealed that such a high level of selenium supplementation was associated with a very high risk (HR = 2.6, CI = 1.44–4.70) of death from prostate cancer compared with nonusers.

The report of the SELECT case–cohort study (1739 men with prostate cancer, of whom 489 were diagnosed with high-grade prostate cancer) [134] demonstrated that selenium supplementation increased the risk of high-grade prostate cancer (by 91%) among men with a high baseline toenail selenium concentration.

**Figure 2:**
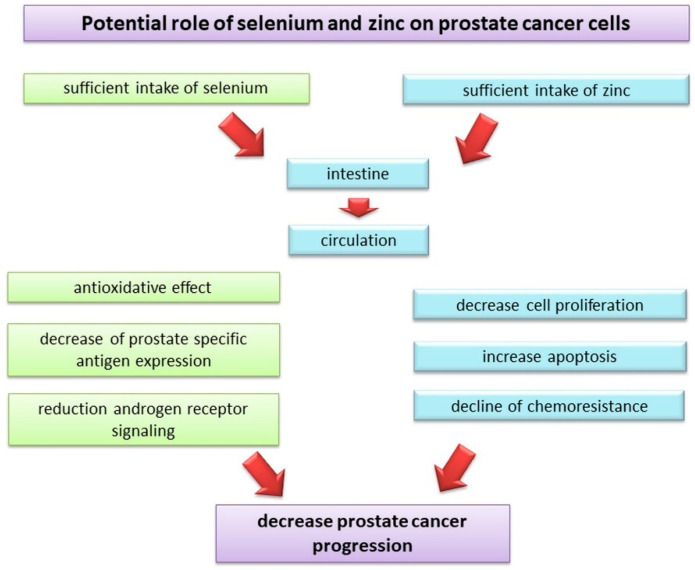
Potential role of selenium and zinc on prostate cancer progression.

Multiple mechanisms (including anti-inflammatory, antioxidative, and androgen receptor-mediated pathways) may be responsible for the protective effect of selenium in relation to the prostate. Metabolites of selenium reduce prostate-specific antigen (PSA) expression and androgen signaling in an androgen-responsive LNCaP prostate cancer cell line [135,136]. Considering the link between chronic prostate inflammation and the induction of prostate carcinogenesis [137], it is also important to pay attention to the anti-inflammatory potential of selenium. Kim et al. [138] revealed that Wistar rats with induced chronic bacterial prostatitis demonstrated lower inflammatory cell infiltration to the prostate following selenium treatment (2 µg/g b.w. daily for 4 weeks) compared to control animals. Moreover, chronic inflammation induced by sexually transmitted infections can lead to ROS-mediated DNA instability of prostate epithelial cells and subsequent neoplasia transformation [139]. Selenium is a component of the active glutathione peroxidase (GPx) center and is considered to be one of the key elements in antioxidant reactions. The role of this enzyme is to maintain the balance between free oxygen radicals, hydrogen peroxide, and organic peroxides that cause damage to cell DNA. GPx, which forms a glutathione complex through selenium, prevents the formation of free radicals because of the formation of hydrogen peroxide (H_2_O_2_), which is the main source of reactive oxygen anions. Thus, this enzyme protects proteins, cell organelles, lipid membranes, and nucleic acids from oxidative stress, thereby preventing deformation and genetic damage to the cells [140] (Figure 2). An increased intake of selenium in the body supports its protective effects, obtained by inducing oxidative stress in cancer cells. This results in their apoptosis under the influence of glutathione. Induction of apoptosis may also result from DNA fragmentation and the activation of numerous caspases under the influence of methylselenol, which is one of the most important metabolites of this trace element [141]. The intensity of apoptosis and growth inhibition is particularly strong in androgen-dependent prostate cancer cells. Laboratory studies have shown that the cytotoxic effect is not observed when selenium acts on normal cells [142]. At appropriately high concentrations, selenium metabolites may also slow down the cell cycle and inhibit protein synthesis, which results in cell growth inhibition. It may also exhibit detoxification activity by bonding to carcinogens, thereby, preventing their activation and subsequent DNA damage.

Other mechanisms of selenium action include the ability to modulate the immune response. Selenium deficiency causes attenuation of immune function, manifested by disturbances in cellular response and B lymphocyte proliferation [143,144]. Selenium supplementation leads to the stimulation of immune response and inhibition of inflammation during carcinogenesis [144].

The recommended daily zinc intake for an adult man is 11 mg [145], and the most essential dietary sources of this mineral element are meat and meat products, cereals and grains, milk and dairy products, and fruits and vegetables (to a lesser extent) [146]. Only 10% of ingested zinc is absorbed in the intestine and enters systemic circulation. The remaining 90% is stored in bones and muscles [147]. As zinc plays an important role in organisms (i.e., antiviral immune response, interaction with selenium and copper, antagonistic action against cadmium and lead, and interaction with cellular signaling molecules) [147], zinc deficiency is associated with central nervous system disorders, liver diseases, hypogonadism, certain types of cancers, and heart diseases (e.g., dyslipidemia) [148].

The results from a Swedish prospective cohort study [149] demonstrated that a high dietary zinc intake was associated with improved survival in patients with newly diagnosed prostate cancer. The study with 525 men diagnosed of prostate cancer (a median period of follow-up was 6.4 years) reported that a high dietary zinc intake (in the range of 15.6–20.1 mg/day) was associated with a 36% lower likelihood of prostate-cancer-specific mortality (HR = 0.64, 95% CI = 0.44–0.94) compared with men who reported a lower zinc intake (from 9.0 to 12.8 mg/day). In addition, when zinc intake was stratified by the prostate cancer stage at the moment of diagnosis, the survival analysis revealed a more protective effect of higher zinc intake in men who were diagnosed at an early stage of prostate cancer (HR = 0.24, 95% CI = 0.09–0.66). However, when prostate cancer was diagnosed at the advanced stage, any significant effect of higher zinc intake was not observed. In turn, the results from the MCC-Spain case–control study [150] covering 733 men with prostate cancer and 1228 population-based control individuals revealed that the dietary zinc intake at the level of at least 10.53 mg/day vs. 8.34 mg/day or less was associated with a 39% higher risk of prostate cancer (OR = 1.39, 95% CI = 1.00–1.95). When zinc intake was compared with prostate cancer aggressiveness, it turned out that patients with a low-grade tumor (Gleason score = 6) more frequently consumed zinc at a higher level (relative risk ratio = 1.66, 95% CI = 1.07–2.57) than patients who consumed less than 8.34 mg/day.

The need for diet supplementation with zinc, especially in men in risk groups, remains open to controversy. The results of a case–control study and meta-analysis by Mahmoud et al. [151] did not show a significant relationship between zinc supplementation and a lower risk of prostate cancer. On the other hand, important findings regarding the relationship between the intake of zinc from supplements and prostate cancer risk have been reported from the Health Professionals Follow-Up Study [152]. Considering the 587,444 person-years of follow-up (a total group of 46,974 men and 2901 prostate cancer cases), a significantly higher relative risk of advanced prostate cancer was revealed in men who consumed more than 100 mg of zinc per day compared to non-users (RR = 2.29, 95% CI = 1.06–4.95). At the same time, supplemental zinc intake below 25 mg/day was associated with a lower relative risk of advanced cancer compared to non-users (age-adjusted RR = 0.75, 95% CI = 0.56–0.99). In addition, the duration of supplemental zinc intake negatively affected the risk of advanced cancer development. The men who consumed zinc from supplements for more than 10 years were at a significantly higher risk (RR = 2.37, 95% CI = 1.42–3.95) of prostate cancer.

Several in vitro and in vivo studies have confirmed the beneficial role of zinc in the chemoprevention of prostate cancer. Xue et al. [153] in an in vitro study with PC3 and DU145 cell lines observed that the anti-proliferative and pro-apoptotic effects of zinc (at a concentration of 200 µM) served as an adjuvant to a first-line chemotherapeutic agent (paclitaxel) and increased the sensitivity of prostate cancer cells to the drug. Similar results were also reported by Zhang et al. [154] in a PC3 cell line. The mechanism of zinc-mediated chemosensitivity of cancer cells proposed by the authors is linked to a direct effect on the induction of apoptosis through the mitochondrial pathway (caspase-3 activation and the release of cytochrome c). It should also be noted that the concentration of zinc in the prostate under physiological conditions is much higher (800–1500 µM) [155] than that in other tissues and rapidly decreases (approximately 60–80%) in the pathological state. Accumulation of zinc in prostate cells allows one to maintain a high concentration of citrate, which is an important component of seminal fluid. It was confirmed that the decline in zinc concentration in malignant prostate cells is due to the downregulation of functional zinc uptake transporters (ZIP1), which is typical for these pathological cells [155]. Considering this aspect, zinc supplementation may be a useful tool in the decline of resistance of malignant cells to chemotherapy (Figure 2). Zinc deficiency can also promote further carcinogenesis by causing an increase in cellular oxidative stress markers (due to the downregulation of antioxidative enzymes), the overexpression of the p53 protein (a key regulator of the cell cycle that allows the cells to repair their DNA damage), and the induction of the generation of single-strand breaks in DNA [155]. Additionally, an experiment conducted by Fong et al. [156] on middle-aged Sprague-Dawley rats maintained on a zinc-deficient diet demonstrated an induction of prostate carcinogenesis on the molecular level.

An interesting issue is an interaction between selenium and zinc in prostate cancer risk modulation. Although the combined effect of zinc and selenium supplementation is largely unknown, it seems that the overall effects depend on the zinc to selenium ratio. Yildiz et al. [157] proposed that the impairment of zinc homeostasis by an increased intake of selenium may contribute to the downregulation of metallothionein proteins that control the intercellular zinc homeostasis and are responsible for the normal oxido-reductive state in prostate cells. Consequently, the downregulation of the p53 protein, decreased apoptosis, and a decline in DNA repair can be observed, which contributes to a higher risk of prostate cancer induction [157]. Additionally, Daragó et al. [158] showed an antagonistic effect of selenium (2.8 µg Se/kg b.w.) and zinc (5 mg Zn/kg b.w. for 90 days) administration to rats on sex steroid hormone levels and androgen receptor (AR) protein expression in the prostate.

#### 3.1.5. Nutrients in Dairy Products

Many studies have shown that significant consumption of dairy products, especially whole milk, is associated with an increased risk of prostate cancer, but this relationship is still not clear. An important aspect is that dairy products are a source of calcium, whose increased intake is considered to be a prostate cancer risk factor [159]. Calcium ions are known to be a key regulator of cell growth, division, and differentiation, and disturbance in calcium homeostasis due to increased calcium intake and lower vitamin D intake may be a significant factor for prostate cancer development [160].

Existing research regarding a link between milk and dairy products intake and prostate cancer risk show conflicting results. The recently published overview of systematic reviews and meta-analyses [161] with cohort studies (involving 848,395 subjects, 38,107 prostate cases, and 4–28 years of follow-up) and case–control meta-analyses (with 12,435 subjects) demonstrates inconsistent results, despite the fact that some of the analyzed reports have proved a positive relationship between a high intake of milk and dairy products and prostate cancer risk. Downer et al. [162] demonstrated higher prostate cancer-specific mortality in men who developed prostate cancer at stage T1-T2 or M0 and consumed high-fat milk for at least 3 servings vs. below 1 serving per day (HR = 6.1, 95 CI = 2.1–17.4). The results of the Prostate, Lung, Colorectal, and Ovarian Cancer Screening Trial showed that an increased consumption of low-fat dairy products was poorly correlated with an increased prostate cancer risk, but an increased calcium intake was associated with an increased prostate cancer risk. It is important to note that the results did not show this relationship for calcium supplementation. Moreover, calcium intake was not related to plasma concentrations of vitamin D [163].

The results concerning the relationship between vitamin D and prostate cancer risk present conflicting results, depending on many factors (e.g., the participant’s age at the moment of diagnosis, the stage of the disease, the measured parameters, etc.). The analysis of 19 prospective studies [164] with data from 13,462 prostate cancer patients and 20,261 control individual participants aimed at the evaluation of prostate cancer risk in relation to circulating vitamin D (25-hydroxyvitamin D and 1,25-dihydroxyvitamin D). The results did not confirm the hypothesis that higher vitamin D concentration contributes to a lower risk of prostate cancer. The authors have found that more circulating 25-hydroxyvitamin D was linked to a higher risk of non-aggressive prostate cancer (OR per 80th percentile increase = 1.24, 95% CI = 1.13–1.36).

Results of in vitro and in vivo studies demonstrated that calcitriol is involved in the inhibition of tumor development by inhibiting the angiogenesis, differentiation, and modulation of tumor-associated growth factor expression, and by cell cycle inhibition. The results of epidemiological studies in most cases indicate that vitamin D3 intake and concentration in the body are important not only as an etiological factor but also as a prognostic factor for various cancers, including prostate cancer. An increased risk of prostate cancer was observed in people with lower levels of vitamin D3, i.e., in the population living in the areas of northern latitudes with little sunlight [165]. This may result from an increased IGF-1 secretion and decreased hormonal synthesis of vitamin D3. Both these factors cause an increase in the proliferation of prostate cells, leading to prostate cancer [166]. Studies on patients diagnosed to have prostate cancer showed that the risk of general and prostate-cancer-related mortality decreases with increasing blood levels of this vitamin [167]. Shahvazi et al. [145] conducted a meta-analysis of clinical trials and showed that vitamin D3 supplementation has no effects on either PSA levels or prostate cancer mortality rates and is not beneficial for patients with this cancer. The authors also observed that it might increase the risk of overall mortality, even though this correlation was not statistically significant. A justifiable mechanism is that vitamin D supplementation increases IGF-1 concentration, which is consistent with the hypothesis that IGF-1 may increase the risk of prostate cancer [145]. Vitamin D metabolites are also essential for proper calcium-phosphate balance and proper bone mineralization. During low calcium concentration, vitamin D3 increases its absorption from food and causes the resorption of bones and calcium release from them in order to maintain a constant plasma level of this vitamin. The antagonistic action of calcium against vitamin D and its derivatives increases the risk of prostate cancer in the case of a high intake of dairy products that are a rich source of this element [161,168].

Some prospective studies also report that the consumption of high-fat milk by individuals with diagnosed prostate cancer favors the risk of recurrence. Such findings were presented by Tat et al. [169] following a Cancer of the Prostate Strategic Urologic Research Endeavor study with 1334 men diagnosed with nonmetastatic prostate cancer (eight years of follow-up). The authors demonstrated that more than four servings of high-fat milk weekly, vs. 0–3 servings monthly, was associated with a significantly higher risk of recurrence (HR = 1.73, 95% CI = 1.00–2.98). In addition, the body mass index (BMI) of patients who consumed whole milk (≥27 kg/m^2^) strengthened (nearly three-fold) the risk of recurrence. In addition, Tarfadottir et al. [170], in a retrospective cohort AGES-Reykjavik Study (with 2268 participants, of whom 1078 were diagnosed with prostate cancer, with a follow-up period of 24.3 years), also reported that milk consumption at least once a day (vs. less than once a day) in early life (before puberty) was associated with an increased risk of prostate cancer at an older age (OR = 1.58, 95% CI = 1.14–2.18). In addition, the age-adjusted and multivariate model adjusted for lifestyle and other dietary factors revealed that a higher milk intake in the adolescence period was associated with a more than three-fold higher risk of advanced prostate cancer at an older age (OR = 3.22, 95% CI = 1.25–8.28). Childhood or adolescent milk consumption may influence the risk of prostate cancer later in life through elevated IGF-1 levels in the critical period of prostate development during puberty. A high intake of calcium may further increase this risk by reducing the levels of circulating 1,25-dihydroxyvitamin D, which is a possible inhibitor of prostate carcinogenesis. Finally, a high intake of animal fat may be associated with increased testosterone levels, which may influence prostate cancer risk. It is worth noting that milk and dairy products are a common source of vitamin A in the diet of people consuming a non-plant-based diet [146]. However, the results regarding the role of retinol in prostate cancer risk are inconsistent. According to Watters et al. [171], the serum retinol level (a marker of vitamin A intake) in patients with prostate cancer was not associated with better survival. In contrast, Hada et al. [172] reported a positive correlation between serum retinol concentration and prostate cancer risk.

### 3.2. Phytonutrients

#### 3.2.1. Vitamin E and Tocopherols

The main sources of vitamin E are vegetable fats, animal products including eggs, and butter. The results of a meta-analysis of prospective nested case–control studies (with eight studies and data from approximately 300,000 subjects) conducted by Cui et al. [173] showed that α-tocopherol concentration in blood was negatively correlated with prostate cancer risk, and that risk decreased by 21% for every increase of blood α-tocopherol concentration by 25 mg/L. Due to the significant heterogeneity among the studies describing γ-tocopherol levels, the association of its blood concentration and prostate cancer risk was not significant [173]. Moreover, the results of another study [174] demonstrated that higher plasma concentrations of both tocopherols in men with prostate cancer recurrence were negatively correlated with serum prostate-specific antigen (PSA) concentration. This observation suggests that a higher intake of food rich in tocopherols might have a positive effect on the serum PSA level in patients with a risk of recurrence. However, previously published results from an α-Tocopherol, β-Carotene Cancer Prevention Study cohort [175] including 29,097 men, of whom 1732 participants were diagnosed with prostate cancer (with a follow-up of up to 19 years) have shown that serum α-tocopherol affects the risk of prostate cancer. The patients with a serum α-tocopherol concentration at the level of at least 14.2 mg/L (compared with those with a concentration lower than 9.3 mg/L) had a lower (by 42%) risk of advanced prostate cancer. Interestingly, the protective relationship was stronger when vitamin E was delivered from supplemental than dietary sources. In addition, Albanes et al. [176] showed that tocopherols can react with selenomethionine, which, when combined with high concentrations of α-tocopherol, may increase the risk of prostate cancer. Taking this into account, particular attention should be paid to the potential higher risk of prostate cancer when supplementing a diet with vitamin E and selenium simultaneously.

The special role of vitamin E involves the prevention of oxidation reactions of PUFAs embedded in the phospholipids and glycolipids of cell membranes and cell organelles. Strong antioxidant effects of tocopherols are observed at the initiation stage of oxidative processes as well as at the propagation stage, in which it interrupts the lipid auto-oxidation chain by the products of their oxidation-free peroxide lipid radicals. The effect of its action involves a reduction in the activity of 3-hydroxy-3-methylglutarylenzyme A (HMG-CoA), which is altered in tumor cells, and the metabolites of its pathway contribute to malignant cell proliferation [177]. γ-Tocopherol and its metabolites also play an important role in the prevention of prostate cancer. Despite its weaker antioxidant effect compared to that of α-tocopherol, it was observed that γ-tocopherol is the strongest lithophilic antioxidant involved in the inactivation of reactive nitrogen forms [178]. γ-Tocopherol and its metabolites inhibit cyclooxygenase activity, thus, modulating inflammatory processes, which may affect carcinogenesis [179].

Vitamin E is an essential component of the systemic antioxidant system. Together with vitamin C, which participates in its regeneration, it more effectively prevents lipid peroxidation. Acting at a high partial oxygen pressure, vitamin E interacts with β-carotene, which is active at a low pressure. It acts synergistically with selenium, supporting the activity of GPx. In addition to its strong antioxidant properties, vitamin E can also prevent carcinogenesis by the non-antioxidative inhibition of cell proliferation and the reduction of protein kinase C activity. Compounds with biological activity of vitamin E have the ability to penetrate the nuclei of cells, where they bind to DNA and prevent rupture and mutation of nucleic acid chains, which counteracts the neoplastic transformations of the cells. Vitamin E can also induce apoptosis through its involvement in the cell cycle. It was also shown that these compounds can inhibit the growth of cancer cells by reducing the concentration of steroid hormones, mainly androgens (testosterone and DHT), in the prostate gland [140].

#### 3.2.2. Selected Flavonoids (Isoflavones, Catechins, and Resveratrol and Its Analogs)

Isoflavones, catechins, and resveratrol and its analogs are flavonoids, which are compounds with high biological activity characterized by a phenolic structure and include more than 4000 chemical compounds. They are found at high concentrations in many fruits and vegetables, mainly in pepper, apples, onions, soya, cereals, and beans, and in tea, especially green tea [180,181]. Some of the substances belonging to this group are metabolized by intestinal microflora to phytoestrogens with a structure similar to that of the human hormone 17β-estradiol. Flavonoids have been shown to increase the mRNA expression of tumor suppressor genes, which are silenced during cancer development. Hence, there is great interest in understanding the mechanisms of anti-cancer activity of these phenolic compounds [182].

##### Isoflavones

The reasons for the lower incidence of prostate cancer in the Asian population include a higher, customary consumption of soya products, which are a source of isoflavones (e.g., genistein and daidzein) [183]. The ability of these compounds to reduce the size of neoplastic foci has also been demonstrated, so they are also active in the progression phase of the disease, reducing its severity, which is reflected in a reduced mortality among patients who consume large quantities of soya and soya products [184]. The results of a meta-analysis by Yan and Spitznagel [185] including cohort and case–control studies on soy (14 publications) and dietary isoflavones consumption (8 publications) showed a significant relationship between increased soybean/isoflavone consumption and reduced (by 26%) prostate cancer risk (95% CI = 0.63–0.89). Analysis of reports shows that non-fermented soy products consumption reduces the risk of prostate cancer even more (combined OR/RR = 0.70, 95% CI = 0.56–0.88). The intake of fermented soy food was unrelated with the risk of the disease. Van Die et al. [186] demonstrated interesting findings in a meta-analysis of randomized, placebo-controlled trials concerning the efficacy of soy or soy isoflavone supplements, providing from 60 to 107 mg of isoflavones or 30 to 450 mg of single phytoestrogens daily. It was demonstrated that soy isoflavone intake was associated with a reduction in prostate cancer diagnosis in men with a clinically confirmed risk of prostate cancer (risk ratio = 0.49, 95% CI = 0.26–0.95), but no significant results were demonstrated regarding the relationship between the intake of soy isoflavones and the serum PSA level in men with confirmed prostate disease. Hwang et al.’s meta-analysis of five cohort and eight case–control studies (with 87,844 participants/1206 prostate cancer cases and 4018 subjects with disease/4407 controls, respectively) [187] revealed that a higher consumption of non-fermented soybean products was significantly associated with a reduced (by 25%) risk of prostate cancer. In turn, a higher total soy food intake decreased the risk of prostate cancer by 31%. At the same time, the most recently published meta-analysis [188] of 30 articles with case–control (15), cohort (8), and nested case–control (7) studies with 266,699 subjects and 21,612 prostate cancer cases reported a lower risk of prostate cancer associated with daidzein and genistein intake (pooled RR = 0.84, 95% CI = 0.0.73–0.97 and RR = 0.90, 95% CI = 0.84–0.97, respectively).

Isoflavones accumulate in the prostate gland where they exhibit a cytotoxic effect on cancer cells [188]. Genistein and daidzein inhibit the growth of prostate cancer cells and reduce the activity of certain enzymes, e.g., 5α-reductase and aromatase, involved in the metabolism of steroid hormones. These compounds may also block the synthesis of PSA by up to 50% [189]. The results of in vivo and in vitro experiments indicate that isoflavones exert anti-cancer effects through multiple mechanisms (e.g., the regulation of androgen receptor transcription, the inhibition of translocation of androgen receptors to the nucleus, and the inhibition of testosterone synthesis and conversion of testosterone to DHT), leading to the inhibition of cancer cell growth and proliferation [190]. Isoflavonoid activity also increases plasma concentrations of SHBG, thus, reducing the pool of free testosterone in serum [190]. Metabolism of cancer cells changes due to their intensive proliferation. Stimulation by androgens causes an increase in glucose uptake as androgens stimulate anabolic processes. The results of an in vitro experiment showed that the incubation of cancer cells with flavonoids (genistein, phloretin, and apigenin) inhibits the growth of these cells, especially in cells sensitive to hormones [191].

##### Green Tea Polyphenols (Catechins)

Green tea polyphenols are also widely studied because of their chemo-preventive action on various cancers, including prostate cancer [192]. The results of previous studies showed that this effect is related to the high content of catechins, such as epigallocatechin-3-gallate and theaflavins, which belong to the group of flavonoids.

The recently published meta-analysis from Filippini et al. [193] based on three randomized controlled studies investigating the effects of green tea extract supplementation reported weak evidence of a lower risk of prostate cancer (RR = 0.50, 95% CI = 0.18–1.36). In turn, the results obtained from five cohort and eight case–control studies with 127,239 participants and 2926 cases demonstrated a lower risk of prostate cancer following increased green tea consumption (RR = 0.73, 95% CI = 0.56–0.94), but significant (*p* = 0.0001) heterogeneity between analyzed publications was found.

Another meta-analysis [194] including four cohort, three randomized controlled trials, and three case–control studies (with 96,332 participants and 1435 prostate cancer cases) on the relationship between the consumption of green tea and disease risk and three randomized controlled studies investigating the relationship between epigallocatechin-3-gallate intake and disease risk (enrolling 179 participants with high-grade prostate cancer or atypical small acinar proliferation) has shown a weak, protective effect of increased green tea consumption on prostate cancer risk. A dose–response analysis demonstrated a reduced risk of prostate cancer with a higher consumption of green tea (more than seven cups/day). In addition, when analyzing three randomized trials of green tea catechin consumption (supplements were taken at a daily dose of 400–600 mg for 12 to 30 months), a protective association with a decreasing risk of prostate cancer was found (RR = 0.38, 95% CI = 0.16–0.86).

The anti-cancer properties of tea polyphenols are related to the inhibition of proliferation and angiogenesis or induction of apoptosis [195]. In vitro experiments with the PC-3 prostate cancer line showed a decreased expression of Bcl-2 and Ki-67, a higher expression of proapoptotic caspases (caspase-7 and caspase-3), and an inhibition of cancer cell metastasis [196,197,198].

##### Resveratrol and Its Analogs

Resveratrol (3,4′,5-trihydroxystilbene) is a phytoalexin found in many plants, including nuts, grapes, apples, red fruits, black olives, capers, and red rice. Stilbene compounds confer plants with resistance to microbial and fungal infections [199]. Resveratrol presents in red wine, and the skin of red grapes has been linked with the “French paradox”, which is the phenomenon of an unexpectedly low rate of heart disease in French people who consume a high amount of red wine, despite the high intake of SFAs [200,201]. The compound has been shown to exert anti-inflammatory and antioxidative effects and affect the initiation and progression of many diseases, including cancer [202,203].

In a literature review (2007–2013) on the role of resveratrol in prostate cancer, Jasiński et al. [204] identified 39 studies on the cell lines and animal models of prostate cancer. The anti-cancer potential of resveratrol has been well documented in many of these studies. The downregulation of the androgen receptor, synergy with flutamide, and the enhancement of radiosensitivity are the most interesting targets in the treatment of prostate cancer. Resveratrol has shown chemo-preventive potential against prostate cancer in both in vitro and animal model studies.

Recent in vivo and in vitro studies have also confirmed the previously mentioned observations. For instance, Jang et al. [205] reported that resveratrol could be used to suppress the metastasis of prostate cancer. In addition, Hsieh et al. [206] found that it can suppress the migration of epithelial prostate cancer cells by attenuating the control of epithelial-to-mesenchymal transition. Ye et al. [207] and Wang et al. [208] reported that resveratrol suppresses the proliferation of prostate cancer cells, promotes apoptosis, and inhibits the expression of androgen receptors. In vivo studies [209,210] demonstrated that a combination of curcumin and resveratrol exhibited strong modulatory potential against prostate carcinogenesis, while a combination of quercetin and resveratrol may serve as a potential novel regimen for the prevention and/or treatment of prostate cancer.

To date, results from human clinical trials on the therapeutic effects of resveratrol in prostate diseases have not been reported. Moreover, although a consistent body of literature on the protective effects of resveratrol against cancer exists, only a few studies have focused on its possible toxicity and adverse effects. Therefore, there is a need for future studies on the long-term effects and in vivo adverse effects of resveratrol supplementation in humans. Considering the above aspects, data on the interactions of resveratrol when combined with other therapies are still lacking, along with results related to its absorption and bioavailability in the human body [211].

Several analogs of resveratrol (e.g., methylated analogs, dimer resveratrol gnetin C, pterostilbene, and piceatannol) have been shown to act against prostate cancer in in vitro, in vivo, and epigenetic studies, and their effects are found to be mediated through different mechanisms [212]. Lundqvist et al. [213] demonstrated, using prostate cancer (LNCaP) and normal prostate (RWPE) epithelial cells, that resveratrol and its analogs inhibited the activation of androgen receptors. Furthermore, Kumar et al. [214] showed in two lines (DU145 and PCM3) of human prostate carcinoma cells that dimer resveratrol gnetin C, which is a natural resveratrol dimer found in melinjo (*Gnetum gnemon* L.) seeds [215], downregulated the metastasis-associated 1 protein (MTA1)-mediated pathway, while promoting the apoptosis and decreasing the aggressiveness of prostate cancer cells. The anti-cancer action mediated by gnetin C turned out to be more potent compared to resveratrol. Li et al. [216] also reported the MTA1-mediated induction of p53 acetylation in prostate cancer cells treated with pterostilbene, which is a natural compound found among others in blueberries and acts as a natural epigenetic factor. Moreover, the authors reported the inhibition of tumor growth, progression, and metastasis in mice intraperitoneally treated with pterostilbene (50 mg/kg/day for eight weeks). Another mechanism related to the anti-cancer action of stilbenes is cell cycle regulation. Kido et al. [217] showed that prostate cancer epithelial (LNCaP) cells treated with (10–40 µM) piceatannol extract from yellow passion fruit significantly arrested the G0/G1 phase of the cell cycle as well as induced p53 protein expression. In addition, based on an in vivo study (TRAMP model mice treated by gavage with 20 mg piceatannol/kg b. wt. for 4 or 10 weeks), the authors demonstrated delayed progression of prostate cancer mediated by decreased expression of cell cycle regulatory proteins (cyclin-dependent kinase 4, cyclin D1, and p21).

**Figure 3:**
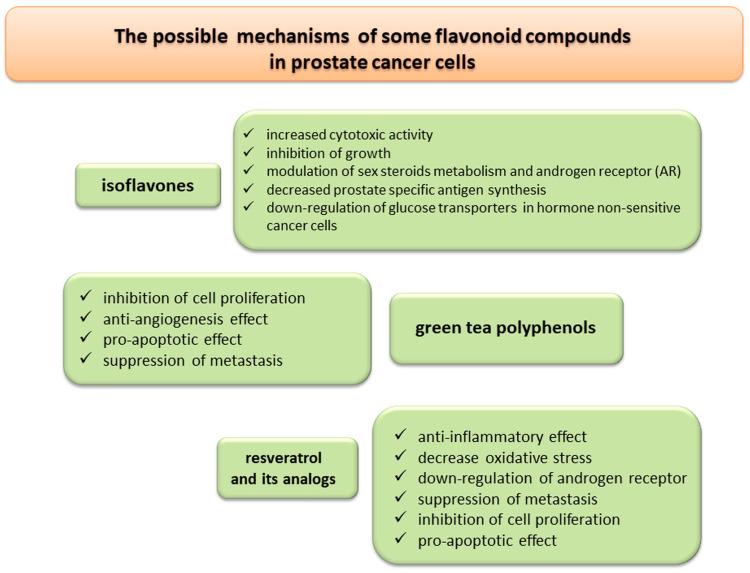
Possible mechanisms of some flavonoid compounds on prostate cancer cells.

The flavonoids-mediated inhibitory effects on prostate cancer development are presented in Figure 3.

#### 3.2.3. Lycopene

Lycopene is an antioxidant carotenoid derived not only from tomatoes but also from tomato sauce, tomato pasta, tomato juice, and ketchup. Its bioavailability is higher after thermal treatment. Therefore, tomato-based products (e.g., sauces) are included as sources of lycopene with high availability. Others fruits (red and pink grapefruits, papaya, goji berries, and melon) and vegetables (red carrot and watermelon) are also sources of dietary lycopene. As a long-chain unsaturated compound containing a system of conjugated double bonds, lycopene exhibits strong antioxidant properties. This pigment can neutralize reactive oxygen species and organic free radicals produced during the peroxidation process. These properties result from the structure and affinity of lycopene to the lipid bilayer in biological membranes [218].

A recently published systematic review and meta-analysis [219] including 42 publications, 25 of which investigated the impact of dietary lycopene and 18 of which were focused on the circulating lycopene in relation to prostate cancer risk. The total number of individuals was 692,012 participants and 43,851 cancer cases. The authors reported that dietary lycopene intake and circulating concentration were significantly related to a lower risk of prostate cancer (RR = 0.88, 95% CI = 0.78–0.98 and RR = 0.88, 95% CI = 0.79–0.98, respectively). It was also stated, based on dose–response analysis, that the additional consumption of lycopene (by 2 mg/day) was significantly associated with a lower (by 1%) risk of prostate cancer. A cohort study conducted by Zu et al. [220] showed that lycopene consumption was more strongly associated with prostate cancer in the pre-PSA stage than in the PSA stage. Moreover, healthy men who consumed more lycopene were reported to have a 28% lower risk of developing lethal prostate cancer than those who consumed less.

The results of an in vitro study on the effect of lycopene on cellular adhesion and migration, which are very important features of cancer progression, show that this carotenoid causes a 40% reduction in the motility of cancer cells [221]. Simone Elgass et al. [221] showed in an in vitro study that a high concentration of lycopene (1.15 μmol/L) inhibits the adhesion and migration of metastatic prostate cancer cells.

Anti-cancerogenic properties of lycopene are associated with numerous mechanisms of its action on the body and can be observed at all stages of carcinogenesis: initiation, promotion, and progression. Lycopene has strong antioxidant properties and is even 10 times more effective in neutralizing free radicals than vitamin E and twice as effective as β-carotene. Thus, despite having a 160-fold lower concentration in prostate cells as compared to the concentration of β-tocopherol, lycopene significantly contributes to the protection of proteins and lipids against oxidation processes. The activity of lycopene in these processes may be observed at different stages. This compound can prevent ROS from reacting with biologically active compounds and interrupt free radical chain reactions and non-radical oxidation processes [222].

Other mechanisms by which lycopene exerts protective effects on the prostate include modulation of proteins’ activity that regulate the cell cycle, the ability to repair DNA damage caused by oxidation reaction, the influence on gene expression in the early phase of carcinogenesis, modulation of enzymes’ activity involved in carcinogen detoxification, and the influence on the biological activity of IGF-1 and the protein binding this hormone (IGFBP-3), which is a metabolic reservoir of IGF-1, prolonging its half-life. Lycopene acts as an inhibitor of IGF-1, which is an identified risk factor for prostate cancer, promoting the processes of proliferation and differentiation of cancer cells. Normal, physiological prostate cells are less sensitive to lycopene. Thus, they can undergo processes of proliferation and growth stimulated by IGF-1 [223,224]. This was confirmed in the study conducted on a group of patients with diagnosed prostate cancer undergoing surgery. A smaller tumor size, inhibition of the metastatic phase of the cancer, and a decrease in serum PSA concentration were observed in patients supplemented with lycopene [225,226].

## 4. Conclusions

Dietary patterns based on processed meat and fatty dairy products, due to their high content of saturated fatty acids and trans fatty acids, as well as a diet low in vegetables and fruits, due to the insufficient amount of vitamins and minerals, are conducive to the development of prostate cancer through a number of mechanisms that stimulate cancer cell proliferation and angiogenesis processes. On the other hand, dietary patterns, based on low-processed plant products and fish, may have a beneficial effect on prostate metabolism and inhibit all stages of carcinogenesis through multiple mechanisms. Promotion of a healthy diet is a key element in preventing prostate cancer.

## Data Availability

Not applicable.
